# Prognostic and predictive value of a lncRNA signature in patients with stage II colon cancer

**DOI:** 10.1038/s41598-022-25852-5

**Published:** 2023-01-24

**Authors:** Ailin Qu, Qian Wang, Qing Chang, Jingkang Liu, Yongmei Yang, Xin Zhang, Yanli Zhang, Xiaoshi Zhang, Hongchun Wang, Yi Zhang

**Affiliations:** 1 Department of Clinical Laboratory, Qilu Hospital, Shandong University, Wenhua Xi Road, Jinan, 250012 Shandong Province People’s Republic of China; 2 Department of Gastroenterology, Central Hospital, Shandong First Medical University, Jinan, 250011 Shandong Province People’s Republic of China; 3 Department of Gynecology, Qilu Hospital, Shandong University, Jinan, 250012 Shandong Province People’s Republic of China; 4 Department of Clinical Laboratory, Shandong Provincial Third Hospital, Jinan, 250031 Shandong Province People’s Republic of China

**Keywords:** Cancer models, Gastrointestinal cancer

## Abstract

The current staging method is inadequate to identify high-risk recurrence patients with stage II colon cancer (CC). Using a systematic and comprehensive-biomarker discovery and validation method, we aimed to construct a lncRNA-based signature to improve the prognostic prediction of stage II CC. We identified 1,377 differently expressed lncRNAs by analyzing 16 paired stage II CC tumor tissue and adjacent normal mucosal tissue from the TCGA dataset. Subsequently, using a univariable and step multivariable Cox regression model, we trained an 11-lncRNA signature in the training cohort (*n* = 141), which could divide patients into high-risk and low-risk groups (AUC at 3 years = 0.801, 95% CI: 0.724–0.877; AUC at 5 years = 0.801, 95% CI: 0.718–0.885). Significantly, patients in the high-risk group had poorer recurrence-free survival (RFS) compared with the low-risk group (log-rank test, *P* < 0.001 in the training cohort). This lncRNA-based signature was further confirmed in the validation cohort (*P* < 0.001). Multivariate Cox regression and stratified survival analyses showed that the prognostic value of this signature was independent of other clinicopathological risk factors (CEA, T stage, and chemotherapy). Time-dependent receiver operating characteristic (ROC) analysis demonstrated that this signature had better prognostic ability than any other clinical risk factors or single lncRNAs (all *P* < 0.05). A nomogram was constructed for clinical use, which integrated both the lncRNA-based signature and clinical risk factors (CEA and T stage) and performed well in the calibration plots. Altogether, our lncRNA-based signature was an independent prognostic factor and possessed a stronger predictive power compared with the currently used clinicopathological risk factors when predicting the recurrence of patients with stage II CC. Collectively, this lncRNA-based signature might facilitate individualized treatment decisions and postoperative counseling, ultimately contributing to improved survival.

## Introduction

Colon cancer (CC) is a common malignancy with substantial mortality worldwide. Approximately 25% of CC patients are diagnosed with stage II disease^1^. About 15–25% of these stage II patients suffer from fatal recurrence (local relapse and distant metastasis), causing poor prognosis and even death^2,3^. Traditionally, most national societies identify high risk of stage II CC patients as those having at least one of the following clinicopathological features: T4 stage, poor histological differentiation, bowel perforation or obstruction, less than 12 lymph nodes examined, lymphovascular invasion, and microsatellite instability (MSI)^4–8^. However, these risk factors can neither identify patients with high recurrence risk nor predict those who benefit from adjuvant chemotherapy^9–11^. Therefore, it is urgently necessary to develop a reliable prognostic and predictive staging approach to identify the true high-risk population of stage II CC patients.

Recent advancements in genome-wide sequencing have provided the extensive landscape of the mammalian genome, including non‐coding RNA. Long non-coding RNA (lncRNA) is a subclass of non-coding RNAs covering > 200 nt in length^12^. They are reported to participate in multiple biological functions, including translation, transcription, splicing, and cellular processes^12,13^, often serving as a competing endogenous RNA (ceRNA) to regulate the expressions of miRNAs and thereby targeting downstream molecules of these miRNAs^14^. Emerging studies have revealed that the aberrantly expressed lncRNAs in tumor tissues play crucial roles in tumorigenesis, proliferation, and metastasis, affecting the prognosis for CC patients^15–17^. These data indicate that the lncRNAs can be potential diagnostic and prognostic biomarkers in CC. Recent findings on lncRNAs in CC also support the development of biomarkers for the precise evaluation of cancer progression^18–21^. However, no comprehensive study on prognostic biomarkers has been carried out based on the expression profiles of lncRNAs in stage II CC patients.

The combination of multiple variables rather than just a single biomarker can provide more robust and accurate information for prognosis, contributing to individualized treatment in this clinical setting^22,23^. In the current study, we conducted a systematic analysis and developed a novel lncRNA-based signature to predict individualized recurrence in stage II CC patients. We initially identified the differentially expressed lncRNAs (DElncRNAs) in paired stage II CC from The Cancer Genome Atlas colon adenocarcinoma (TCGA-COAD). Then, the DElncRNAs were subjected to univariable and step multivariable Cox regression analysis to train a lncRNA-based signature to predict recurrence-free survival (RFS) in stage II CC patients. Finally, the lncRNA signature was validated and incorporated into a prognostic nomogram. Additionally, we compared its predictive performance with other clinicopathological risk factors.

## Materials and methods

### Ethical statement

All procedures about human participants were in accordance with the ethical standards of the Clinical Research Ethics Committee of Qilu Hospital, Shandong University and performed in accordance with the Declaration of Helsinki. Understanding and written informed consent were obtained from each subject.

### Patients and clinical database

The enrolled patients of this study were from the publicly available TCGA dataset and a clinical validation set from Qilu Hospital, Shandong University. In the TCGA cohort, transcriptome profiling information and corresponding clinical pathological data of stage II colon patients were downloaded from https://portal.gdc.cancer.gov. The gene transfer format (GTF) files (Homo sapiens.GRCh38.91.chr.gtf) from Ensemble (http://asia.ensembl.org) were used to annotate the data and distinguish mRNAs and lncRNAs.

Patients with lack of survival information and less than one month follow -up time were excluded, and as a result, 141 stage II colon cancer patients were included. Among them, 16 patients with paired tumor and adjacent normal tissues were used to screen differentially expressed lncRNAs. Then 141 stage II colon cancer patients were used as the training set. In the clinical validation set, we collected 63 formalin-fixed paraffin-embedded (FFPE) samples of stage II CC in Qilu Hospital, Shandong University (Jinan, China) between October 2009 and September 2013 based on the following criteria: (a) pathological confirmed colon cancer with stage II disease (T3-4, N0, M0); (b) with related clinical pathological information and survival data; (c) none of the patients have received preoperative chemotherapy, radiotherapy or chemoradiotherapy; (d) without other tumor diseases meanwhile. All of the specimens were assessed by two pathologists based on the AJCC/UICC TNM grading system 8th edition.

### RT-qPCR analysis of lncRNA expression

We firstly extracted the total RNA from 10-μm-thick FFPE specimens by RNAprep pure FFPE kit (cat. no. DP439; TIANGEN Biotech, Beijing, China). All the process involving RNA were conducted in RNase-free conditions. The cDNA was synthesized from an equal amount of total RNA of each sample using SureScript™ First-Strand cDNA Synthesis kit (cat. No. QP056; GeneCopoeia, Guangzhou, China) according to the manufacturer’s instructions. lncRNA expression was assessed by Bio-Rad CFX96 Detection System (Bio-Rad, Hercules, CA) with Blaze Taq™ SYBR Green qPCR Mix 2.0 (cat. No. QP033; GeneCopoeia, Guangzhou, China). The lncRNA expression levels were calculated using the 2^−dCT^ method with GAPDH as the reference gene. The obtained expression data were then log2 transformed. The primers for all lncRNAs and GAPDH used were purchased from Ribobio (Guangzhou, China), and the primers information was list in Table S1.

### Study procedures

**Figure 1:**
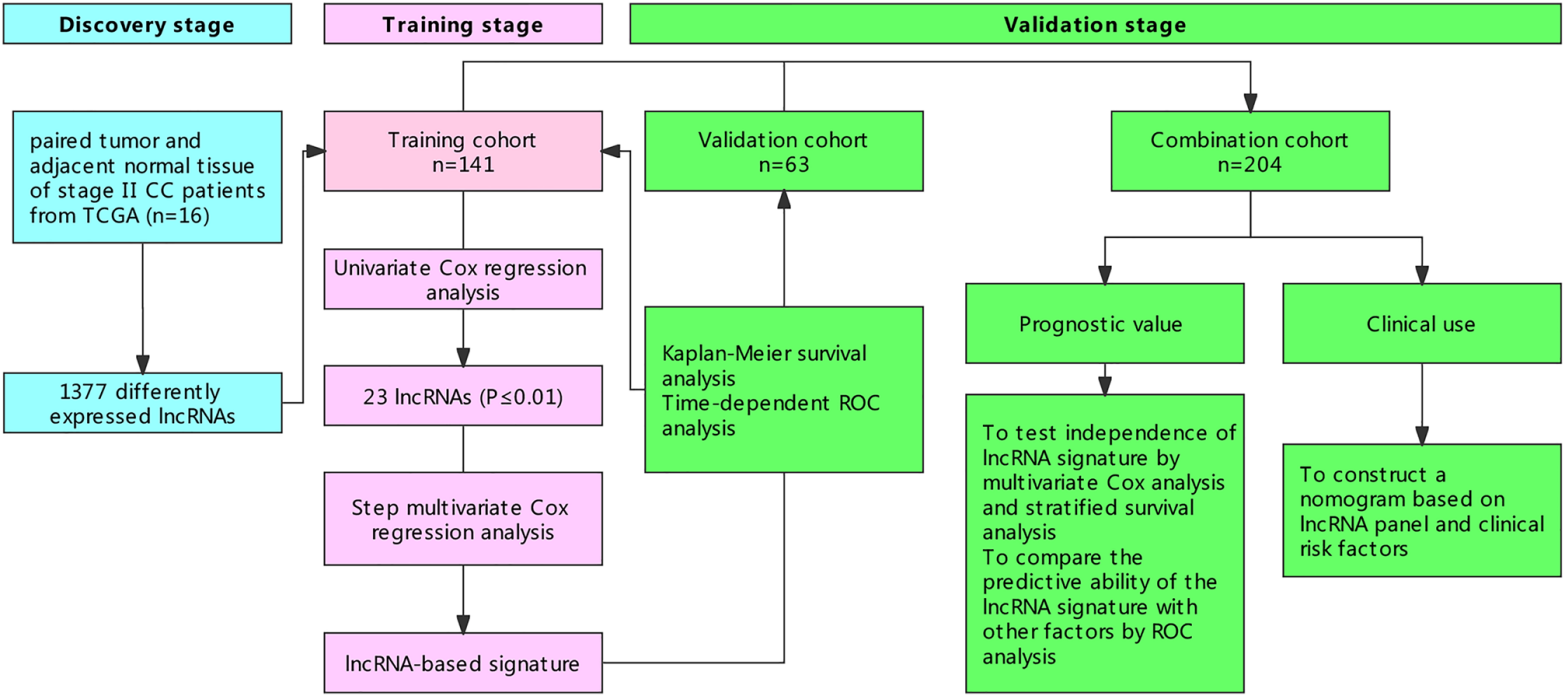
The flow chart of our study design.

This study was performed in three stages: discovery stage, training stage and validation stage. A flowchart of the procedures is shown in Fig. 1. In the discovery stage, 16 paired tumor and adjacent normal tissue of stage II colon cancer patients from TCGA dataset were used to screen differentially expressed lncRNAs. In the training stage, the obtained candidate lncRNAs were entered univariate Cox proportional hazard regression model to evaluate the correlation between lncRNA level and RFS in the training set. Subsequently, the lncRNAs with top statistical significance (*P* value ≤ 0.01) were subjected to a step multivariate Cox regression model to train lncRNA signature. A survival-related model for stage II colon patient was established to predict prognosis which using selected lncRNA expression, weighted by their multivariate Cox regression coefficients as follows: $\mathrm{Riskscore}=\sum_{\mathrm{i}}\mathrm{coefficient}\left({\mathrm{lncRNA}}_{i}\right)\times \mathrm{expression}\left({\mathrm{lncRNA}}_{i}\right)$. X-tile plots (X-tile, version 3.6.1; Yale University School of Medicine, New Haven, CT, USA) was used to obtain the optimum cut-off value), and patients in the training set were divided into high- and low-risk groups. Kaplan–Meier curve and time dependent ROC curve were used to examine the prognostic ability of lncRNA-based signature. In the validation stage, we calculated the risk score of patients in the validation set using the same risk score formula obtained from the training set. Then we divided the patients into high-risk group and low-risk group using the cutoff value from the training set. Kaplan–Meier curve and ROC curve were used to examine the prognostic performance of the lncRNA signature in the validation set.

### Statistical analysis

Statistical analysis and graph plotting were performed by R software (version 3.4.2; http://www.Rproject.org). Statistical significance was set at 0.05. Categorical variables were analyzed using Pearson’s chi-squared test or Fisher’s exact test as appropriate. For survival analyses, we used the Kaplan–Meier method to plot survival curves and used log-rank tests to compare the difference. The univariate analysis and multivariate analysis of prognostic factors were performed using Cox proportional hazard regression model. Time-dependent ROC analysis was applied to examine the prognostic ability (‘survivalROC’ package), and the bootstrapping method with 10,000 iterations was performed to compare the differences between the AUCs. A nomogram was built by using the regression coefficients in multivariable Cox regression model to weigh each variable. Calibration plot and ROC curve were used to assess the performance of nomogram (“rms” package).

## Results

### Clinical characteristics of the enrolled participants

Table 1 shows the detailed clinical and pathological characteristics of the enrolled patients, which were similar between the training and validation cohorts (all *P* > 0.05).

**Table 1:** Baseline characteristics of patients in the study.

	Training	Cohort	*n* = 141	*p*	Test	Cohort	*n* = 63	*p*	P*
	Total	Low risk	High risk		Total	Low risk	High risk		
**Gender**									
Female	64	50 (45.9%)	14 (43.8%)	0.992	28	22 (43.1%)	6 (50%)	0.914	0.998
Male	77	59 (54.1%)	18 (56.2%)		35	29 (56.9%)	6 (50%)		
**Lymphatic invasion**									
No	104	84 (81.6%)	20 (76.9%)	0.798	49	40 (81.6%)	9 (75%)	0.910	0.997
Yes	25	19 (18.4%)	6 (23.1%)		12	9 (18.4%)	3 (25%)		
**Microsatellite instability**									
No	23	22 (81.5%)	1 (50%)	0.876	10	9 (81.8%)	1 (50%)	0.944	0.912
Yes	6	5 (18.5%)	1 (50%)		3	2 (18.2%)	1 (50%)		
***T* stage**									
T3	132	104 (95.4%)	28 (87.5%)	0.231	59	49 (96.1%)	10 (83.3%)	0.331	0.996
T4	9	5 (4.6%)	4 (12.5%)		4	2 (3.9%)	2 (16.7%)		
**Venous invasion**									
No	106	86 (87.8%)	20 (87%)	0.998	49	39 (83%)	10 (83.3%)	0.926	0.551
Yes	15	12 (12.2%)	3 (13%)		10	8 (17%)	2 (16.7%)		
**Age**^**a**^									
Younger	75	57 (52.3%)	18 (56.2%)	0.847	31	25 (49%)	6 (50%)	0.981	0.710
Older	66	52 (47.7%)	14 (43.8%)		32	26 (51%)	6 (50%)		
**LN count**									
Fewer_than_12	16	14 (13.6%)	2 (7.7%)	0.629	8	7 (14.3%)	1 (8.3%)	0.944	0.998
12_or_more	113	89 (86.4%)	24 (92.3%)		53	42 (85.7%)	11 (91.7%)		
**CEA**									
Normal	60	46 (75.4%)	14 (73.7%)	0.996	29	24 (82.8%)	5 (71.4%)	0.883	0.681
Abnormal	20	15 (24.6%)	5 (26.3%)		7	5 (17.2%)	2 (28.6%)		

P* the difference between the training cohort and test cohort. ^a^The average age was 61.

### Identification of DElncRNAs by analyzing the TCGA dataset

First, we retrieved the transcriptome profiling data from TCGA-COAD database and obtained 16 normal samples and 152 tumor samples with stage II CC. Among them, 16 paired tumor tissue and adjacent normal tissue were used to screen DElncRNAs. As a result, 1,377 lncRNAs were identified as DElncRNAs with an absolute fold change > 2 and an FDR < 0.05 (Table S2), among which 863 were upregulated, and 514 were downregulated in CC compared with adjacent normal tissue (Figure S1).

### Identification of the prognostic lncRNAs from the training cohort

**Figure 2:**
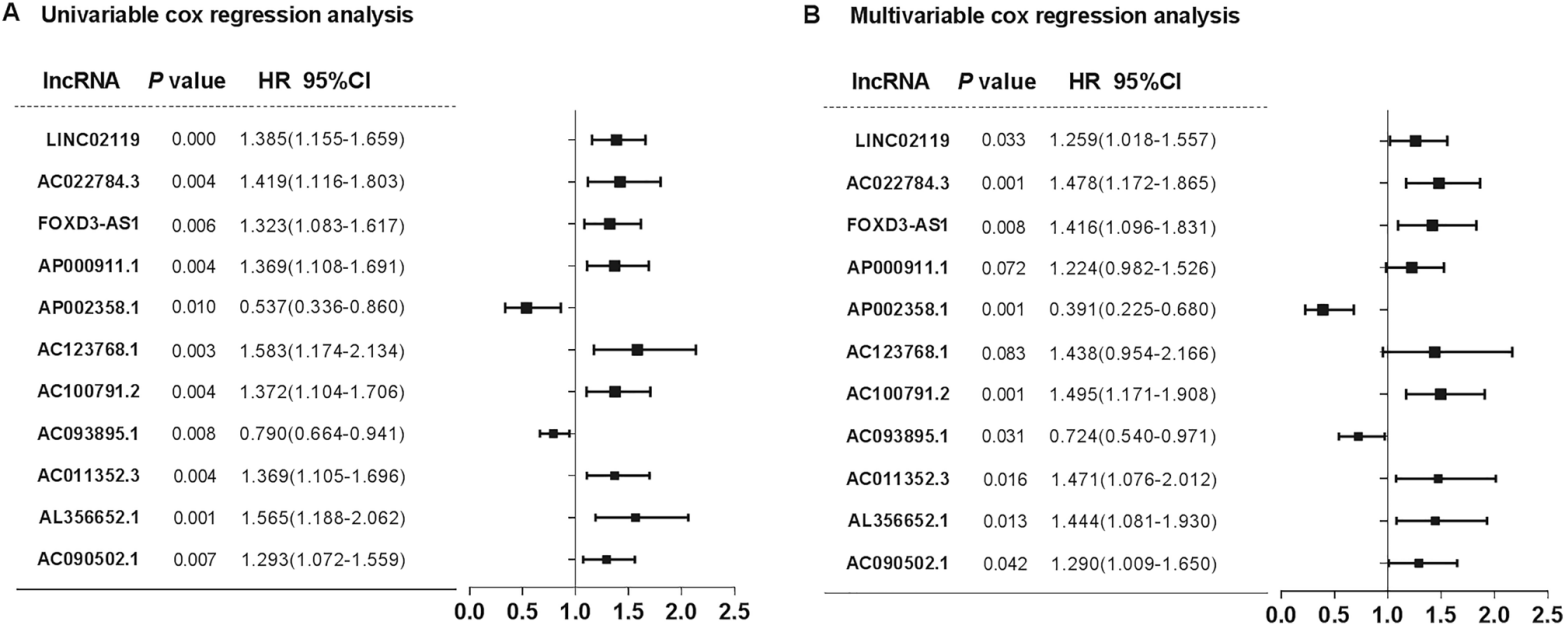
Forest plot summary of analyses of stage II colon cancer patients’ prognosis. Univariate and multivariate Cox regression for the eleven lncRNAs in the training set. The squares on the transverse lines represent the hazard ratio (HR), and the transverse lines represent the 95% confidence interval (95% CI).

To single out the prognostic lncRNAs, the 1,377 DElncRNAs were submitted to the univariate Cox regression analysis to examine their assassination with RFS in the training cohort. Of these DElncRNAs, 23 candidate lncRNAs with top statistical significance (*P* value ≤ 0.01) were entered into a multivariate Cox proportional hazards model by stepwise method (Table S3). As a result, we trained an RFS-related signature consisting of 11 lncRNAs (Fig. 2). Among these lncRNAs, AC090502.1, AL356652.1, AC011352.3, AC100791.2, AC123768.1, AP000911.1, FOXD3-AS1, AC022784.3, and LINC02119 with positive coefficients were identified as risk makers owing to the close correlation between their high expressions and poor RFS of patients, whereas AC093895.1 and AP002358.1 were protective factors.

### Construction of a lncRNA prognostic risk model and its predictability assessment in the training cohort

**Figure 3:**
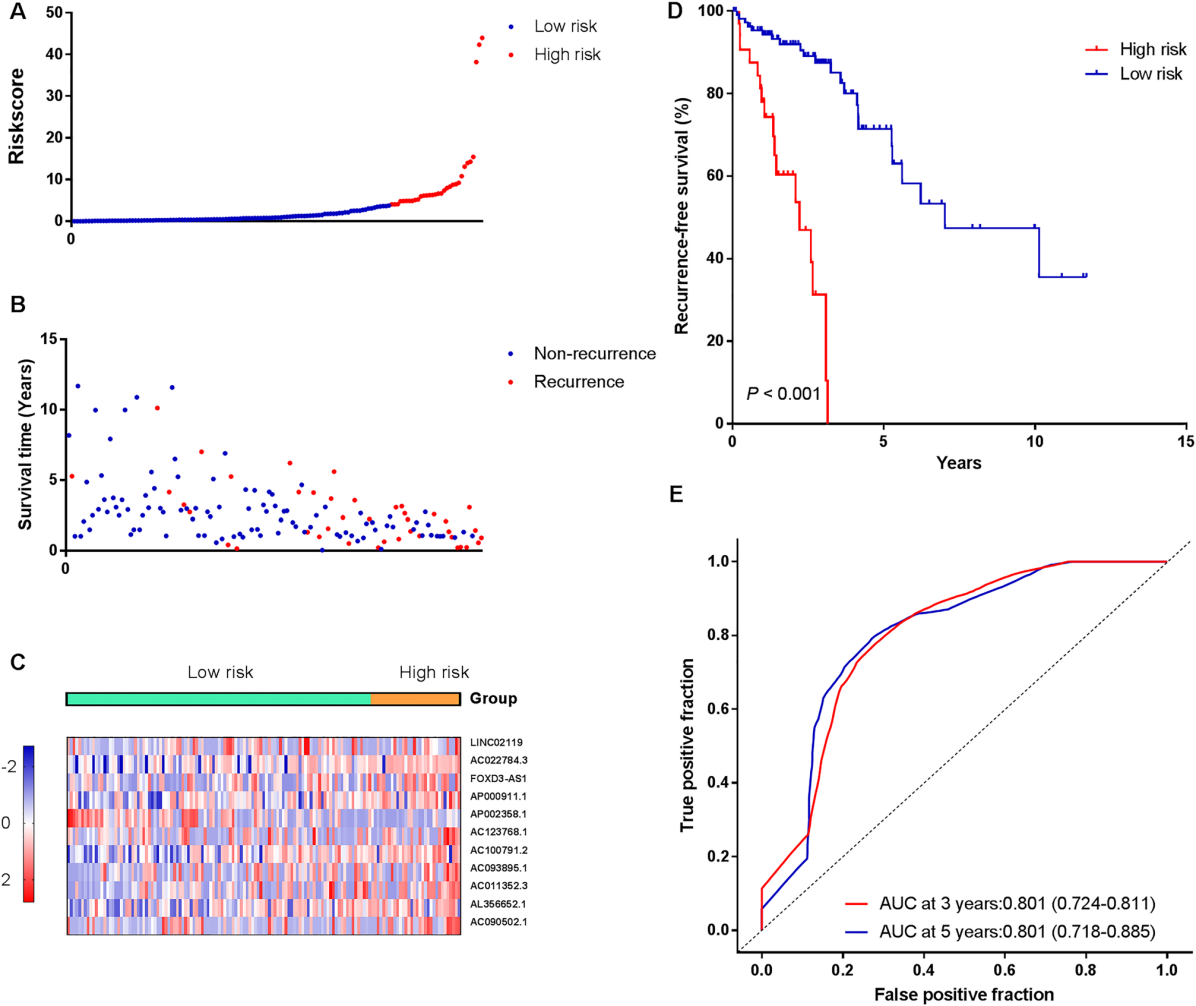
Identification of a 11-lncRNA signature significantly associated with patients’ RFS in the training cohort. (**A**–**C**) Risk score distribution, survival status, and lncRNA expression patterns for patients in high- and low-risk groups by the lncRNA signature. (**D**) Kaplan–Meier curve analysis of patients’ RFS in high- and low-risk group. (**E**) Time-dependent ROC curves analysis. We used AUCs at 3 and 5 years to assess the prognostic accuracy, and calculated *P*-value using the log-rank test.

We used the regression coefficients of the multivariate Cox regression model to weight the expression of each lncRNA in the prognostic lncRNA signature, and a risk score formula was established as follows: Risk score = (0.2549*AC090502.1) + (0.3677*AL356652.1) + (0.3862*AC011352.3) + (-0.3231*AC093895.1) + (0.4019*AC100791.2) + (0.3629*AC123768.1) + (-0.9391*AP002358.1) + (0.2024*AP000911.1) + (0.348*`FOXD3-AS1`) + (0.3906*AC022784.3) + (0.2307*LINC02119). Based on this formula, the risk score of each patient in the training cohort was calculated, and the patients were stratified into two groups: a high-risk group (*n* = 32) and a low-risk group (*n* = 109) according to the cutoff threshold obtained from X-tile plots (Figure S2). Figure 3A,B show the distribution of risk scores and recurrence status, respectively, indicating that high-risk patients generally had poorer survival than low-risk ones. The heatmap showed the expression pattern of lncRNAs between the high-risk and low-risk groups (Fig. 3C). Kaplan–Meier survival curves demonstrated that patients in the high-risk group had a shorter RFS (Fig. 3D) and OS (Figure S3A) compared with the low-risk group (log-rank test, *P* < 0.001). The time-dependent ROC at varying time points showed that the lncRNA signature harbored a promising prognostic ability to predict the recurrence of patients in the training cohort (AUC at 3 years = 0.801, 95% CI: 0.724–0.877; AUC at 5 years = 0.801, 95% CI: 0.718–0.885) (Fig. 3E). In the univariate Cox regression model, the risk of recurrence (95% CI: 4.649–16.482, *P* < 0.001) in the high-risk group was increased by 8.754-fold compared with the low-risk group.

### Validation of the lncRNA signature for RFS prediction in the validation cohort

**Figure 4:**
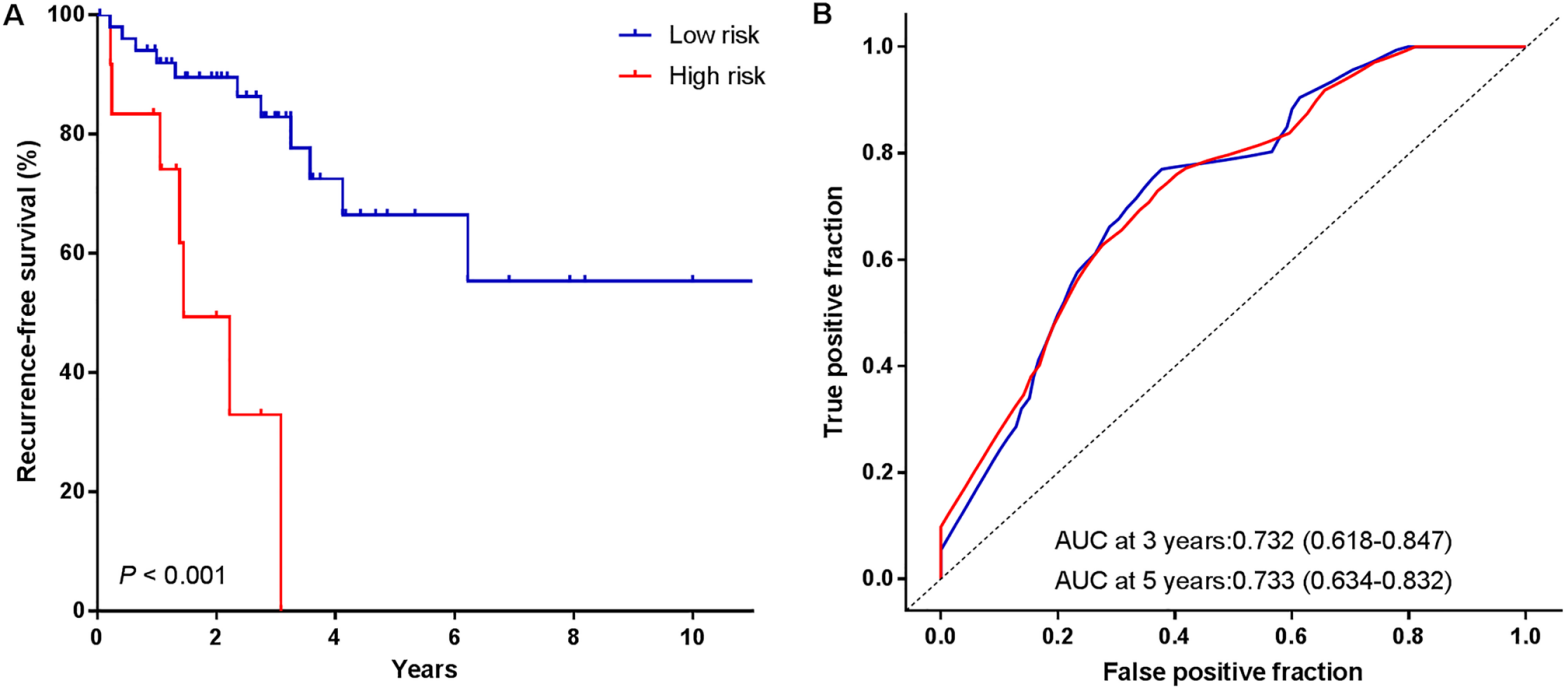
Kaplan–Meier survival analysis and time-dependent ROC curves of the lncRNA signature in the validation cohort. (**A**) Kaplan–Meier curve analysis of patients’ RFS in high- and low-risk group. (**B**) Time-dependent ROC curves analysis. We used AUCs at 3 and 5 years to assess the prognostic accuracy, and calculated *P* value using the log-rank test.

To evaluate the robustness of the lncRNA signature in identifying high-risk patients, we further examined the prognostic performance of the signature using the validation cohort. We calculated the risk score of patients in the validation cohort and divided them into high-risk group and low-risk group. The same survival analysis was performed as in the training cohort. Consistent with the findings of the training cohort, high-risk patients had poorer RFS (Fig. 4A) and OS (Figure S3B) than low-risk patients in the validation cohort. Time-dependent ROC analysis (Fig. 4B) indicated that the AUC for the lncRNA signature to predict the recurrence was 0.732 (95% CI: 0.618–0.847) at 3 years and 0.733 (95% CI: 0.634–0.832) at 5 years, highlighting the validity of the lncRNA signature.

### Prognostic value of the lncRNA signature

**Figure 5:**
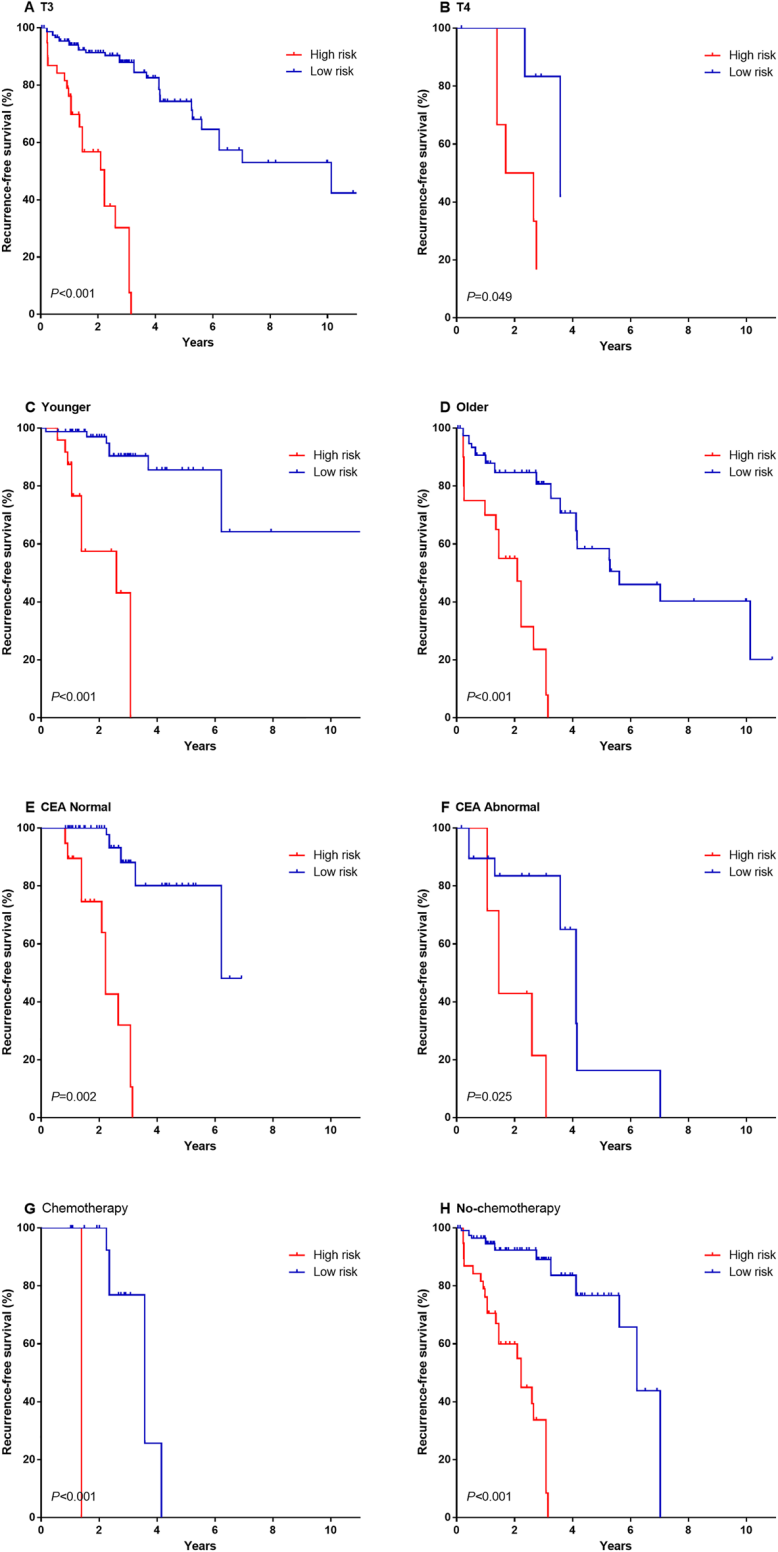
Kaplan–Meier survival analysis according to the 11-lncRNA signature stratified by clinicopathological risk factors in all 204 stage II colon patients. (**A**, **B**) T stage. (**C**, **D**) age. (**E**, **F**) preoperative CEA level. (**G**, **H**) postoperative chemotherapy or not. We calculated *P* values using the log-rank test.

To examine whether the lncRNA signature could predict recurrence irrespective of other clinicopathological features, we performed the univariable and multivariable Cox regression analyses in the entire cohort consisting of 204 patients (combination of the training and validation cohorts). The results indicated that the risk score of the lncRNA signature was significantly correlated with the RFS of patients even when adjusted by other clinical parameters (Table 2). Besides, the age, T stage, and preoperative CEA level of patients were significant prognostic factors in stage II CC patients in univariable analyses (all *P* < 0.05). To better assess the prognostic potential of our lncRNA signature, a stratification analysis was introduced to confirm the independence of our lncRNA signature in various subgroups (according to age, T stage, and preoperative CEA level). Figure 5 shows that the survival curves of the high-risk group were situated below those of the low-risk group in all subgroups. In addition, log-rank tests showed that high-risk patients had poorer RFS compared with low-risk ones in all subgroups (Fig. 5A,B,C,D,E,F). Some stage II CC patients were treated with postoperative adjuvant chemotherapy, which could affect the outcome and recurrence of patients. To eliminate the potentially confounding effect, we also performed stratification analysis by postoperative chemotherapy, and the results showed that high-risk patients identified by the lncRNA-based signature had poorer RFS than the low-risk ones in both chemotherapy and no-chemotherapy subgroups (Fig. 5G,H), confirming its reliable predictive ability regardless of the chemotherapy status.

**Table 2:** Univariate and multivariate Cox proportional hazards analysis of factors associated with RFS in all 204 patients.

Variables	Univariable analysis			Multivariable analysis		
	HR	95% CI	P		95% CI	P
**Gender**						
Male vs. female	1.182	(0.704–1.986)	0.526			
**Age**						
Older vs. younger	2.377	(1.349–4.188)	0.003	1.087	(0.539–2.190)	0.816
**Microsatellite instability**						
Yes vs. no	0.596	(0.130–2.732)	0.505			
**T**						
T4 vs. T3	2.968	(1.381–6.379)	0.005	3.221	(1.405–7.386)	0.006
**Venous invasion**						
Yes vs. no	1.180	(0.527–2.644)	0.687			
**Lymph node examined count**						
12 or more vs. less 12	0.990	(0.467–2.100)	0.979			
**Lymphatic invasion**						
Yes vs. no	1.534	(0.798–2.946)	0.199			
**Post chemotherapy**						
Yes vs. no	1.395	90.654–3.021)	0.397			
**CEA**						
Abnormal vs. normal	2.777	(1.431–5.391)	0.003	2.514	(1.285–4.919)	0.007
**LncRNA signature**						
High risk vs. low risk	8.754	(4.649–6.482)	0.000	10.430	(4.539–3.969)	0.000

**Figure 6:**
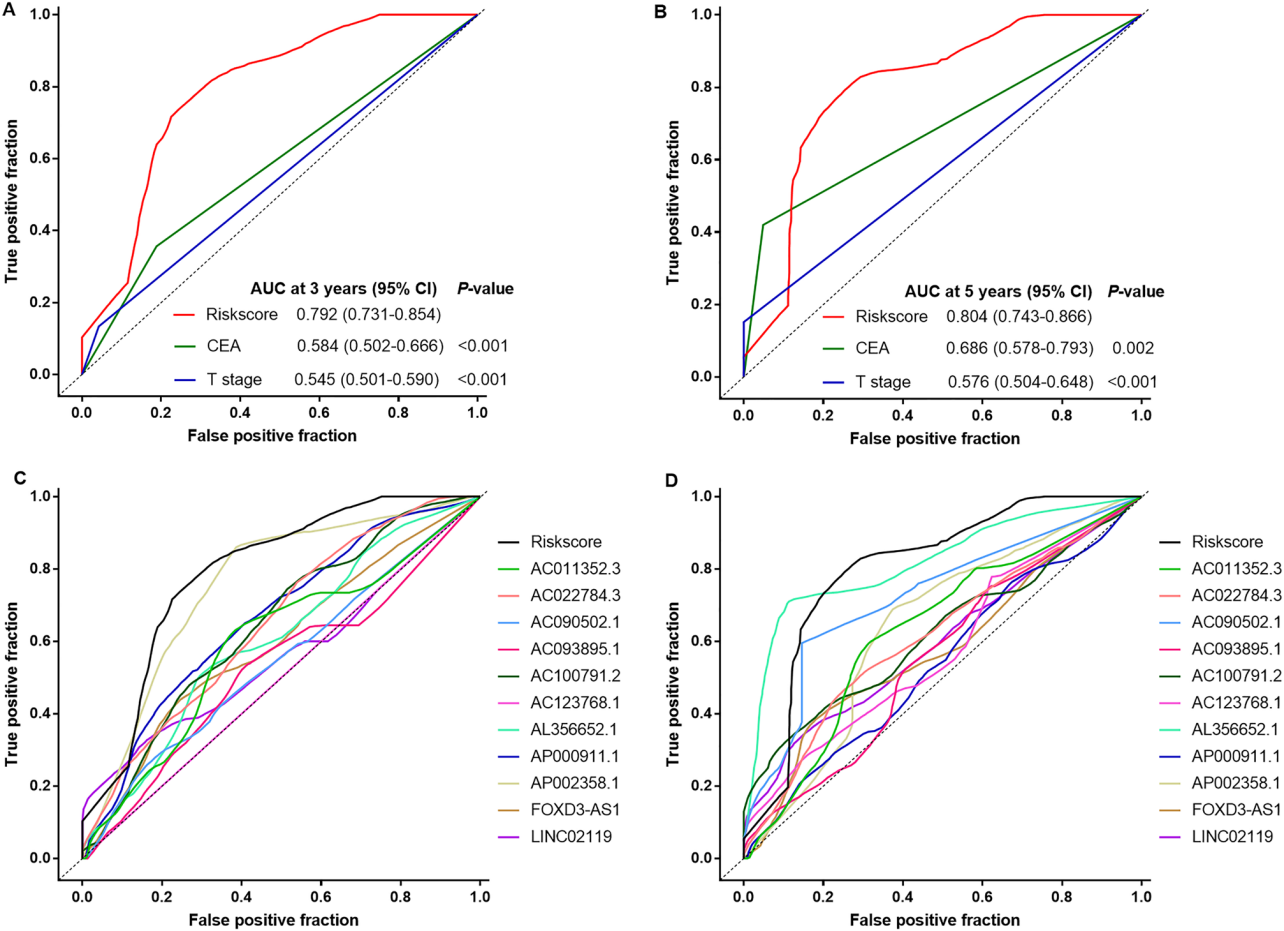
Time-dependent ROC curves to compare the prognostic accuracy of the 11-lncRNA signature with clinicopathological risk factors and single lncRNAs in the combination cohort. (**A**, **B**) Comparisons of the prognostic accuracy by the 11-lncRNA-based signature, age, preoperative CEA level and T stages. (**C**, **D**) Comparisons of the prognostic accuracy by the 11-lncRNA-based signature, and single lncRNA. *P* values show the AUC of the lncRNA signature vs the AUC of other factors.

The multivariable Cox analyses showed that preoperative CEA level and T stage were independent prognostic factors for RFS in patients with stage II CC. We then performed ROC analysis to compare the predictive ability of the lncRNA signature with preoperative CEA level and T stage. Figure 6 shows that the lncRNA-based signature risk score model possessed a more substantial predictive power than any other risk factors (preoperative CEA level and T stage), or single lncRNA alone (all *P* < 0.05), confirming the reliable predictive ability of our lncRNA signature.

### Construction of nomogram based on the lncRNA signature

**Figure 7:**
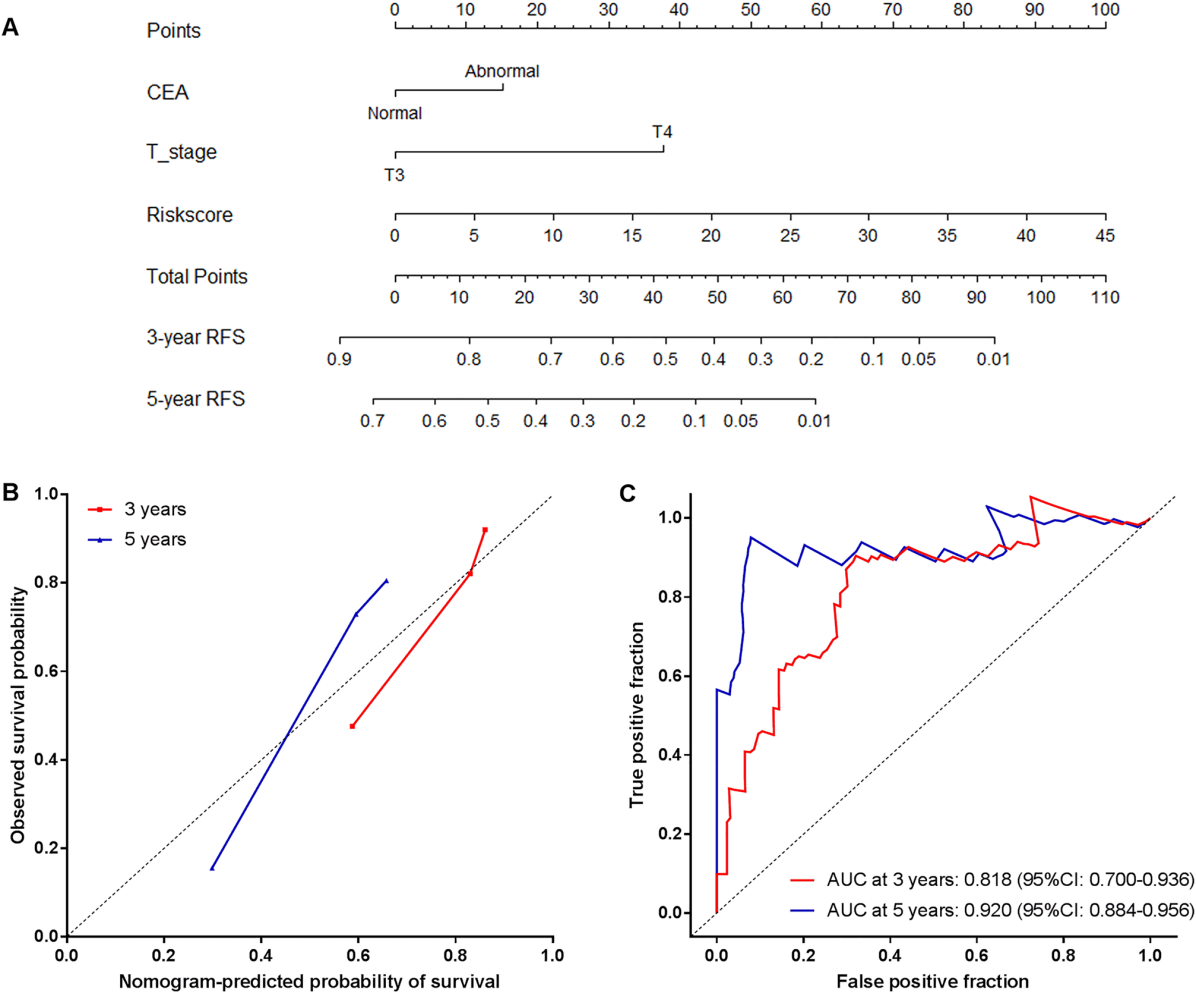
The nomogram to predict probability of RFS for stage II colon patients in all 204 patients. (**A**) The nomogram for predicting proportion of patients with RFS. (**B**) The calibration plots of the nomogram for the probability of RFS at 3 and 5 years. (**C**) Time-dependent ROC based on the nomogram for recurrence probability. Nomogram-predicted probability of recurrence is plotted on the x-axis and observed recurrence probability is plotted on the y-axis.

To provide a quantitative method for the clinician to predict the probability of cancer recurrence, we constructed a nomogram that integrated both the lncRNA signature and clinicopathological independent risk factors for patients’ RFS (including T stage and preoperative CEA level) (Fig. 7A). Calibration plots showed that the bias-corrected lines of 3 and 5 years were very close to the ideal 45-degree curve, indicating high agreement between prediction and observation (Fig. 7B). In addition, the predictive accuracy of the nomogram was assessed through survival ROC analysis. The AUCs of the nomogram at 3 and 5 years were 0.818 (95% CI: 0.700–0.936) and 0.920 (95% CI: 0.884–0.956), respectively, demonstrating a favorable discrimination performance (Fig. 7C).

## Discussion

In the present study, we developed and validated a novel prognostic lncRNA-based signature to predict postoperative tumor recurrence for stage II CC patients. Our results demonstrated that this lncRNA-based signature could successfully divide patients into the high-risk group and low-risk group with significant differences in both RFS and OS. Furthermore, the prognostic and predictive value of this lncRNA-based signature was superior to other clinical risk factors. When stratified by these clinical risk factors, the lncRNA-based signature maintained its strong prognostic value.

The survival of CC patients primarily depends on the stage at diagnosis^6^. Although diagnosed in locoregional disease, stage II CC contributes to 16% of CC-related death^24^. Moreover, it is more heterogeneous than other stages of the tumor, which can be divided into low-, intermediate- and high-risk groups according to the widely recognized clinicopathologic high-risk factors of the National Comprehensive Cancer Network (NCCN) guidelines^5^. Postoperative adjuvant chemotherapy is necessary for stage III patients to preclude recurrence and improve survival^5^. As for most patients with stage II disease, complete surgical resection alone is enough, and adjuvant chemotherapy brings specific adverse effects with a survival improvement of less than 5% at 5 years^7,25,26^. Therefore, it is urgently necessary to identify the minority of stage II patients with high recurrence risk who really benefit from adjuvant chemotherapy. In the present study, we constructed and validated a prognostic lncRNA-based signature to predict recurrence. The signature could effectively stratify patients into high-risk and low-risk groups. The identified high-risk patients were recommended to receive adjuvant chemotherapy after surgery. As a result, reduced recurrence and extended life expectancy were observed. The identified low-risk patients were cured by radical resection alone, thereby avoiding unnecessary adjuvant chemotherapy, as well as its adverse events, cost, and inconvenience.

Previous studies have reported multiple differentially expressed lncRNAs between CC and normal tissues, which play roles in the carcinogenesis and progression of CC^27,28^. In particular, ZEB1-AS1, FAM83H-AS1, LINC01296, and LINC01234 have been reported to be correlated with clinicopathological parameters and patients’ survival^18–20,29^. ZEB1-AS1 is highly expressed in CC, and a high level of ZEB1-AS1 is associated with poor survival in CC patients^18^. As a common aberrant lncRNA in several cancers, FAM83H-AS1 functions by regulating TGF-β signaling and leads to poor CC prognosis^19^. However, these studies focus on single lncRNAs and concern all disease stages of CC rather than specific stage II disease. The multivariate COX proportional hazard regression model helps to combine multiple lncRNAs into one panel, which can significantly improve the prognostic efficiency over single ones. Our team developed a lncRNA-based signature consisting of 11 RFS-related lncRNAs by using the univariate and stepwise multivariate COX method in the TCGA dataset. The signature was validated in another cohort and demonstrated to be an independent prognostic factor, holding better predictive ability than clinicopathological risk factors.

Among the identified 11 lncRNAs, AC090502.1, AL356652.1, AC011352.3, AC100791.2, AC123768.1, AP000911.1, FOXD3-AS1, AC022784.3, and LINC02119 were risk factors, whereas AC093895.1 and AP002358.1 were protective factors. The biological function of some lncRNAs enrolled in our signature has been investigated previously. As a crucial regulatory effector, FOXD3-AS1 is closely associated with multiple types of cancers, including CC^30–33^. Wu and colleagues have found that FOXD3-AS1 up-regulation implies poor survival in CRC patients, which is consistent with our results. They have also explored the underlying mechanism and demonstrated that FOXD3-AS1 can promote the progression of CC by regulating the miR-135a-5p/SIRT1 axis^30^. Guo has reported that FOXD3-AS1 is overexpressed in non-small cell lung cancer, and FOXD3-AS1 upregulation promotes the tumor progression by regulating the miR-135a-5p/CDK6 axis in non-small cell lung cancer^31^. AP002358.1 has been reported to be an essential gene of the enhancer RNA panel, which is closely related to the prognosis of thyroid cancer patients and involved in tumor development. Consistent with our results, they have also suggested that AP002358.1 is a “low-risk factor” for its high level is associated with a good prognosis in thyroid cancer patients^34^. The remaining lncRNAs have not been researched yet. Therefore, further studies are required to explore the contribution and function of these lncRNAs in CC.

In the present study, the combined model consisting of the 11 lncRNAs exhibited a significant association with the survival of CC patients. Multivariate Cox analysis showed that the 11-lncRNA-based signature could predict the recurrence of CC independently of the traditional clinical parameters. Stratification analysis showed that our lncRNA signature could effectively stratify patients into high- and low-risk groups within all subgroups. Time-independent ROC analysis demonstrated that the lncRNA signature possessed a stronger predictive power than other clinical risk factors. Since some stage II CC patients were treated with postoperative adjuvant chemotherapy, this could affect the outcome and recurrence of patients. To eliminate the potentially confounding effect, we examined the association between the 11-lncRNA-based signature and recurrence in both chemotherapy and no-chemotherapy subgroups. The results indicated that high-risk patients identified by the lncRNA-based signature had poorer RFS than the low-risk ones in all subgroups, confirming its reliable predictive ability regardless of the chemotherapy status.

A prognostic nomogram is a visual tool based on Cox proportional hazards regression model. Variables closely related to prognosis are assigned specific values according to their contribution to outcome events (named regression coefficient), and the total scores of all variables are calculated to obtain the individual event probability and realize the individualized prediction of prognosis^35,36^. The prognosis and recurrence of tumors are jointly affected by genes as well as clinicopathological parameters. To maximize the use of patients' clinical information, we constructed a nomogram model based on the aforementioned lncRNA-based signature and independent clinicopathological variables (including *T* stage and preoperative CEA level) to realize the visualization of a complex mathematical formula. The calibration curves and time-dependent ROC curve analysis showed that our nomogram model had a good fitting and favorable prediction accuracy, respectively. Therefore, our nomogram model could serve as an essential tool for risk stratification and prognosis prediction in patients with stage II CC, facilitating individualized treatment decisions and postoperative counseling and ultimately contributing to improved survival.

Collectively, we constructed and validated an RFS-related lncRNA-based signature, which could effectively classify stage II CC patients into low- and high-risk groups for tumor recurrence. Furthermore, the signature was proved to possess reliable prognostic and predictive value for recurrence of patients, which was superior to other traditional clinical risk factors. However, this signature should be further validated in large-scale multi-center clinical trials.

## Supplementary Information

Supplementary Information 1.

Supplementary Information 2.

Supplementary Information 3.

Supplementary Information 4.

## Data Availability

All data generated or analyzed during this study are included in this published article and its Supplementary Information files.
