# Supplementary material for: Prognostic and predictive value of a lncRNA signature in patients with stage II colon cancer
Source: Sci Rep. 2023 Jan 24;13:1350. doi: 10.1038/s41598-022-25852-5 (PMC9873786; doi:10.1038/s41598-022-25852-5)

Figure S1. Volcano plot showing the differentially expressed lncRNAs in 16 paired tumor and adjacent normal tissues of stage II colon cancer patients from the TCGA cohort.

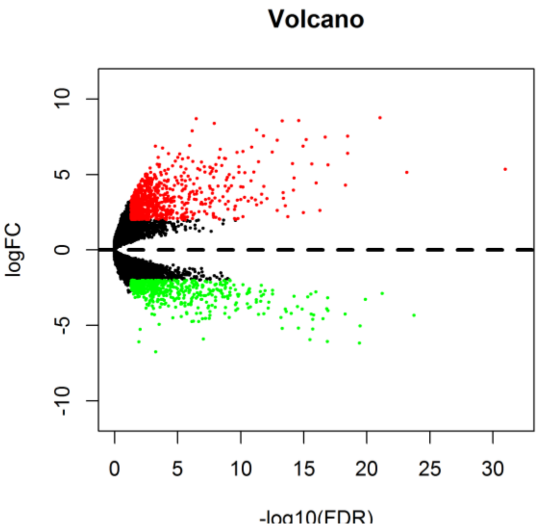

Figure S2. X-tile plots of the lncRNA signature and the risk score in the training cohort.

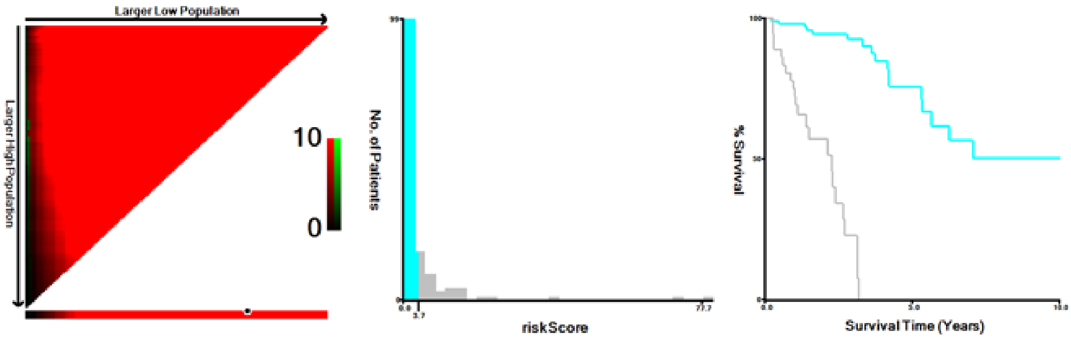

Figure S3. Kaplan-Meier curve analysis of patients' OS in high- and low-risk group in the training cohort (A) and validation cohort (B).

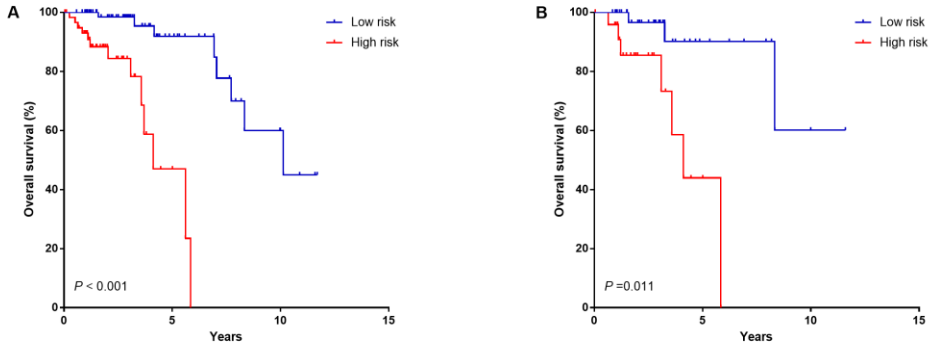

Supplement: Supplementary file 1 — Supplementary Information 1. [file 41598_2022_25852_MOESM1_ESM.pdf]
